# Susceptibility of adults of the cerambycid beetle Hedypathes betulinus to the entomopathogenic fungi Beauveria bassiana, Metarhizium anisopliae, and Purpureocillium lilacinum

**DOI:** 10.1093/jis/14.1.127

**Published:** 2014-09-15

**Authors:** M. E. Schapovaloff, L. F. A. Alves, A. L. Fanti, R. A. Alzogaray, C. C. López Lastra

**Affiliations:** 1 Centro de Estudios Parasitológicos y de Vectores (CEPAVE-CONICET-UNLP). Boulevard 120 S/N e/61 y 62 (B1902CHX). La Plata, Buenos Aires, Argentina; 2 Universidade Estadual do Oeste do Paraná (UNIOESTE). Laboratório de Biotecnologia Agrícola. Campus Cascavel, Paraná, Brasil; 3 Centro de Investigaciones de Plagas e Insecticidas (CIPEIN-CITEDEF/CONICET), Villa Martelli, Buenos Aires, Argentina. Instituto de Investigación e Ingeniería Ambiental, Universidad Nacional de San Martín (UNSAM), Argentina

**Keywords:** biological control, pathogenicity, insect pests, yerba mate

## Abstract

The cerambycid beetle *Hedypathes betulinus* (Klug) (Coleoptera: Cerambycidae) causes severe damage to yerba mate plants ( *Ilex paraguariensis* (St. Hilaire) (Aquifoliales: Aquifoliaceae)), which results in large losses of production. In this study, the pathogenicity of entomopathogenic fungi of the species *Beauveria bassiana* (Balsamo-Crivelli) Vuillemin (Hypocreales: Cordycipitaceae), *Metarhizium anisopliae sensu lato* (Metschnikoff) Sorokin (Hypocreales: Clavicipitaceae), and *Purpureocillium lilacinum* (Thom) Luangsa-ard, Hywel-Jones, Houbraken and Samson (Hypocreales: Ophiocordycipitaceae) on yerba mate were evaluated. Fifteen isolates of *B. bassiana* , two of *M. anisopliae* , and seven of *P. lilacinum* on *H. betulinus* adults were analyzed under laboratory conditions. The raw mortality rate caused by *B. bassiana* isolates varied from 51.1 to 86.3%, and their LT _50_ values varied between 8.7 and 13.6 d. The isolates of *M. anisopliae* caused 69.6‒81.8% mortality, and their LT _50_ values varied between 7.4 and 7.9 d. In contrast, isolates of *P. lilacinum* were not pathogenic. *M. anisopliae* and *B. bassiana* isolates were pathogenic against *H. betulinus* adults, suggesting that they may be useful in biological control programs for insect pests of yerba mate.

## Introduction

Yerba mate plants, *Ilex paraguariensis* (Saint Hilaire) (Aquifoliales: Aquifoliaceae), plantations are attacked by many pests, including the cerambycid beetle *Hedypathes betulinus* (Klug) (Coleoptera: Cerambycidae), which causes severe damage resulting in economical loss in this crop (Casanello 1993). The most severe damage is caused by larval galleries built up or down in the branches and trunks of yerba mate that prevent the normal flow of sap and cause the death of the plant ( Alencar 1960 ). Insecticides, for the control of immature forms and adults, are not recommended because of the risk of toxic residues in the final product ( Borges 2007 ).

In agricultural fields, the entomopathogenic fungal species have been investigated for their potential as the biological control agents because of their role as natural enemies for insects. Conidia that adhere to the surface of the host release extracellular enzymes, including lipases, proteases, and chitinase that help breach the host’s chitinous exoskeleton ( Pendland et al. 1993 , Freimoser et al. 2003 , Tscharntke et al. 2005 ). These fungi have been documented to occur naturally in more than 750 species of hosts and have been used in the development of microbial insecticides ( Hajek and St. Leger 1994 , Inglis et al. 2001 , Shah and Pell 2003 ).

Until now, most of the studies about these fungi have been based on isolation from cadavers of insects or soil (Vu et al. 2007, Abdo et al. 2008 , Glare et al. 2008 ; Santoro et al. 2008 , Brownbridge et al. 2010). In this context, the genera *Paecilomyces, Lecanicillium, Aschersonia, Beauveria,* and *Metarhizium* have been used successfully on experimental or field applications ( Monzón 2001 , Dos Santos and Pozo 2003 , Pucheta et al. 2006 ). In general, fungi are an excellent alternative to conventional pesticides because they can infect different stages of its hosts’ development; they also are nearly pathogenic or not at all to beneficial organisms and humans ( Ferron 1977 ).

Globally, the two most common and studied fungi are the entomopathogenic *Beauveria bassiana* (Balsamo-Crivelli) Vuillemin (Hypocreales: Cordycipitaceae) and *Metarhizium anisopliae sensu lato* (Metschnikoff) Sorokin (Hypocreales: Clavicipitaceae) because they are efficient and easily propagated ( Rodriguez et al. 2006 ). *B. bassiana* is a popular registered mycoinsecticide that has a target list of 700 host insect species (Li 1988, Glare and Milner 1991 , Humber 1991 , Goettel et al. 2000 ). It is ubiquitous in distribution and is pathogenic to a wide spectrum of arthropods; its host range spans most orders of class Insecta (Butt and Goettel 2000 , Lacey et al. 2001 , Zimmerman 2007 ). *M. anisopliae* has a wide host range; it has been documented that they can parasitize more than 300 species of insects of various orders ( Gómez et al. 1997 ).

*Purpureocillium lilacinum* (Thom) Luangsa-ard, Hywel-Jones, Houbraken and Samson (Hypocreales: Ophiocordycipitaceae) is a soil fungus with a good potential for biological control. This species has been described as being as efficient as the commonly used ne-maticides ( Dube and Smart 1987 , Schenck 2004 , Mendoza et al. 2007 , Núñez et al. 2012); it is also a controller of insects ( Posada et al. 1998 , Suh et al. 2002 , Gökçe and Er 2005 , Wakil et al. 2012 ) and others arthropods ( Fiedler and Sosnowska 2007 , Shin et al. 2011 , Angelo et al. 2012 ). According to Bellows (2001) , Headrik and Goden (2001), Lanza et al. (2004) , and other authors, the use of entomopathogenic fungi is an excellent method for the biological control of insects.

The natural occurrence of the fungus *B. bassiana* infecting insects and mites in cultivation of yerba mate ( Ribeiro et al. 1994 , Dalla Santa et al. 2009 ) opens different options for its use to improve phytosanitary plant conditions and the quality and productivity of yerba mate, thereby responding to the current demands of the market ( Borges et al. 2003 ). Leite et al. (2000) isolated a strain of *B. bassiana* CG 716 of adult *H. betulinus* collected in field in Ivaí, PR, Brazil, which has been deposited in the Collection of Embrapa Genetic Resources and Biotechnology. This isolation CG 716 was evaluated under field conditions, demonstrating its potential for control of *H. betulinus* . The importance of selection studies are continually made to be the basis for the success of biological control programs using entomopathogenic fungi ( Alves 1998 , Borges 2007 ). In Argentina, there are no screening studies of native fungal isolates for controlling insect pests of yerba mate. We believe that this is the first research reported about selection of isolates of entomopathogenic fungi for control of *H. betulinus* .

The objective of this study was to determine the pathogenicity of *B. bassiana* , *M. anisopliae* , and *P. lilacinum* isolates on adults of *H. betulinus* under laboratory conditions.

## Materials and Methods

### Insects

*Hedypathes betulinus* adults were collected manually and individually from a yerba mate plantation in Ivaí, Paraná, Brazil. The insects were individualized in plastic containers of 11 × 8 cm, with a perforated lid, and transported to the laboratory. The insects were placed in wooden cages of 60 × 40 × 40 cm and were fed on branches of yerba mate. The cages were kept in controlled room (26 ± 1°C, photoperiod of 14:10 L:D, and 70% RH).

### Fungal isolates

From 24 monosporic isolates, 15 corresponded to the fungus *B. bassiana* , two isolates belonged to *M. anisopliae sensu lato* , and seven to *P. lilacinum* . They were obtained from soil samples collected from different crops of yerba mate plantation in the different locations of Misiones province, Argentina ( Table 1 ). Fungal species were identified according to taxonomic keys ( Samson 1974 ; Samson et al. 1988 ; Humber 1996 , Humber 1997 ; Tzean et al. 1997 ; Hodge 2006 ). The fungal isolates were cultured on Sabouraud dextrose agar complemented with 1% yeast extract (SDAY) in 90 mm Petri dishes and incubated for 10 d at 26 ± 1°C and 14 h pho-tophase. After this period, conidia were collected and stored in glass tubes at ‒10 °C. Fungal isolates were deposited in the Mycological Collections of the Centro de Estudios Parasitológicos y de Vectores (CEPAVE, La Plata, Buenos Aires, Argentina).

**Table 1.**
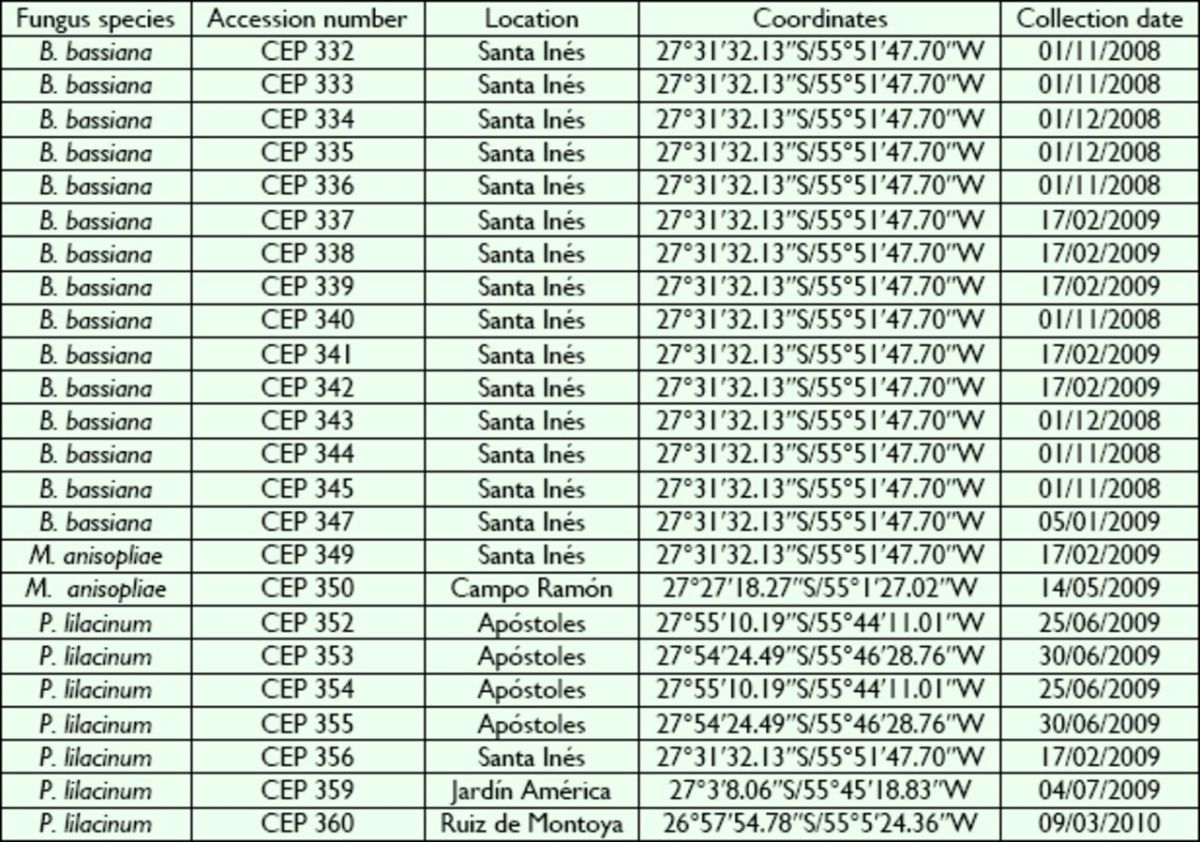
Details about the entomopathogenic fungi collected in the province of Misiones (Argentina) and used in pathogenicity tests against adult *Hedypathes betulinus*

Conidial viability of each isolate was assessed after 24 h using the techniques described by Lane et al. (1988) . Conidia were examined under a microscope at 400× magnification. Conidia were considered germinated when germ tubes were longer than conidial length. A total of 600 conidia were evaluated, and relative percent germination was calculated.

### Bioassays against *Hedypathes betulinus*

A suspension of conidia in 0.01% Tween 80 from fungal sporulated cultures was prepared, and the concentration was adjusted to 1 × 10 ^8^ conidia/mL, based on hemocytometer count. For each isolate, the inoculation was performed by immersing 30 adults insects individually in the conidial suspension for 10 s, then transfering the treated insects to 90 mm diameter Petri dishes lined with filter paper to remove excess suspension of conidia. They were then transferred to plastic cups containing a branch of yerba mate. The cups were closed with a perforated plastic lid. Control insects consisted of adults immersed in sterile distilled water with 0.01% Tween 80. Insects were maintained under controlled conditions as described above. The bioassays were repeated three times. The adults were examined for mortality every 24 h for 15 d. Dead insects were removed and sterilized superficially in 70% alcohol and two successive baths with distilled water. Then they were placed in moist chambers and maintained under the same controlled conditions for the emergence of the mycelium of the fungus. Mortality was confirmed by observing the insects under the stereomicroscope.

### Statistical analysis

In each treatment, the cumulative mortality was corrected for control mortality according to Abbott’s formula ( Abbott 1925 ). Percentage of germinated conidia and percentage of mortality was analyzed by Statgraphics Centurion 15.2 program (StatPoint 2007). An arcsine transformation was performed to stabilize the variance of germination and percent mortality. A test of homogeneity of variance was performed to detect variation between each experiment. Then, data were submitted to analysis of variance (ANOVA) and Tukey’s multiple range tests ( *P* < 0.05). Lethal time 50% (LT _50_ ) values were calculated by using the statistical software for correlated data developed by Throne et al. (1995) .

## Results

Viability of conidia from *B. bassiana* , *M. anisopliae* , and *P. lilacinum* isolates was higher than 89% at 24 h ( Table 2 ). *B. bassiana* isolates caused infection and death of *H. betulinus* adults. Signs of infection were observed by external growing of fungal mycelia through the insect hosts cuticle; the fungal growth was first observed in intersegment membranes at the abdomen, then at mouthparts, antenna, and legs, where they sporulated and produced white conidia. The differences in mortality rate of *H. betulinus* controlled and treated for all 15 isolates used were highly significant ( *F* = 5.77, df = 15, *P* < 0.0001). *B. bassiana* isolates caused > 50% mortality. The highest values were caused by isolate CEP 334, and the lowest was for CEP 347 ( Table 2 ). The LT _50_ values ranged from 8.7 d (isolate CEP 340) to 13.5 d (isolate CEP 345) ( Table 2 ).

**Table 2.**
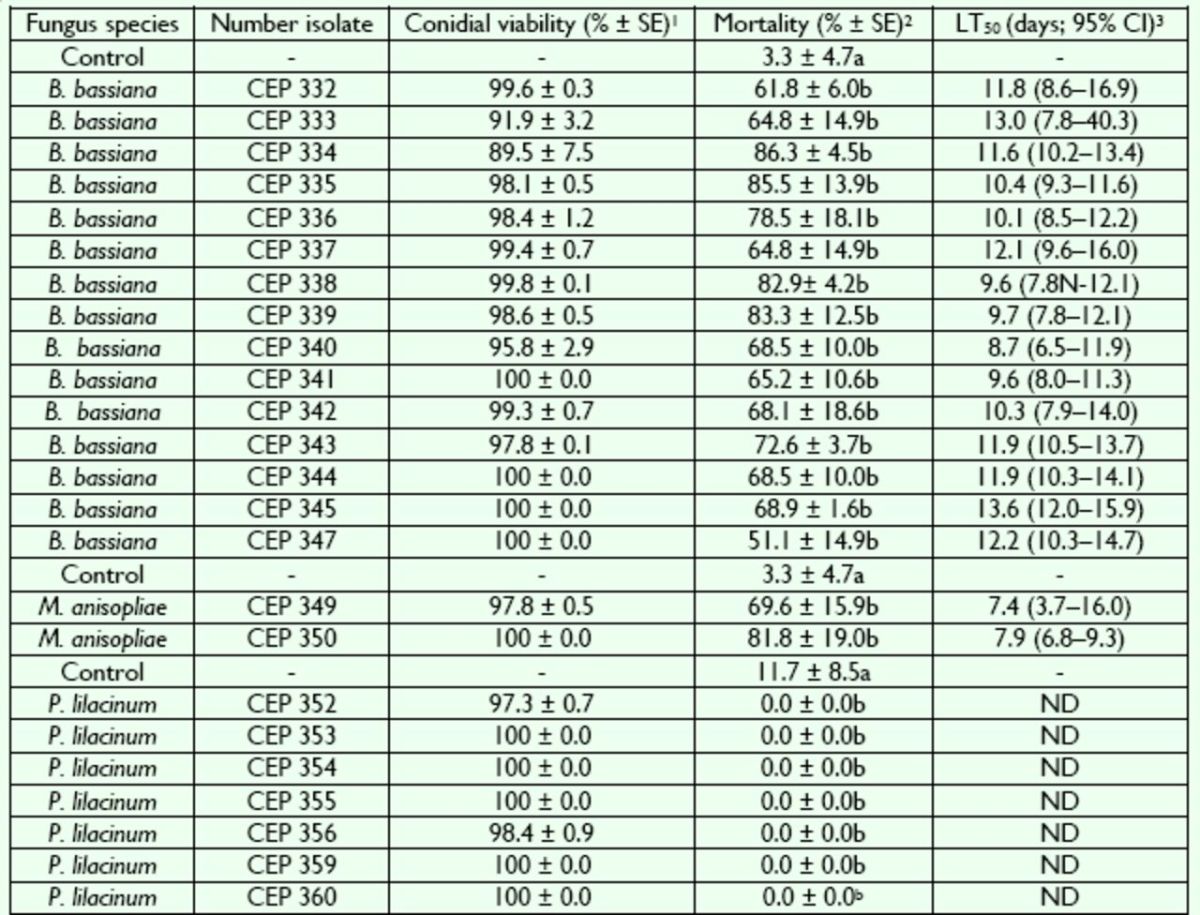
Cumulative mortality and median lethal time (LT50) of entomopathogenic fungi on adult *Hedypathes betulinus*

1Mean percentage of conidial viability (standard error) obtained after incubation at 25°C during 24 h.

2Corrected mortality using Abbott’s formula. Within column, means followed by different letters are highly and significantly different ( *P* ≤ 0.01; Tukey’s test).

395% confidence intervals. ND: date not determinated.

*M. anisopliae sensu lato* isolates caused infection and death of *H. betulinus* after 7 d, and mycelia were observed all over the insect body with olive green color sporulation. The difference in mortality rate between controlled and treated insects was highly significant ( *F* = 16.82, df = 2, *P* = 0.0035). The *M. anisopliae* isolates caused > 60% mortality, the maximum value being for CEP 350 and the lowest for CEP 349 ( Table 2 ). The LT _50_ values ranged from 7.4 d (isolate CEP 349) to 7.9 d (isolate CEP 350) ( Table 2 ).

The seven isolates of P. lilacinum were not infective to H. betulinus. The differences in mortality rate of H. betulinus betwen controlled and treated insects were significant (F = 3.77, df = 7, P = 0.0133). Purpureocilliumlilacinum isolates were not pathogenic. Because of this low mortality, it was not possible to calculate LT50 values ( Table 2 ).

## Discussion

In this study, B. bassiana and M. anisopliae sensu lato isolates presented high pathogenicity against H. betulinus adults. The different isolates of P. lilacinum, however, were not pathogenic. Hafez et al. (1994) reported that infection levels are the result of contact between a virulent strain and an insect cuticle that is susceptible to the fungus conidial germinationand germ tube penetration. Finally, the pathogen can develop inside the insect body.

St. Leger et al. (1992) suggested that insects’ susceptibility or resistance to a particular fungus can be determined by cuticle components at the beginning of the infection. Another aspectto be considered about the increase of pathogenicity is the B. bassiana production of extracellular enzymes in a nutritious culture medium (for example, proteases, lipases, and chitinases), all of which are included in host cuticle penetration and, consequently, in the infection by the fungus; their expression is influenced by cuticle composition and its own genesis in the culture medium ( St. Leger et al. 1992 , El Sayed 1993 ).

Pagliosa et al. (1994) reported similar results to ours when they evaluated B. bassiana CG 152; a mortality rate of 73.4% and a LT50 value of 9.4 d were obtained for adult H. betulinus under laboratory conditions. Shimazu et al. (2002) evaluated the pathogenicity of fungi B. bassiana, M. anisopliae, and Paecilomyces (= Isaria) sp. against the Cerambycidae Anoplophora glabripennis (Mots). The fungi were applied on larvae in a concentration of 1 × 107 conidia/mL. B. bassiana F0003 was the most infective isolate, causing 100% mortality with an LT50 value of 16 d.

Other previous reports of Beauveria sp. For control of the yerba mate pests Thelosia camina Sachus (Lepidoptera: Bombycidae) and Hylesia Hub. (Lepidoptera: Saturniidae) produced between 15.9 and 97% mortality when insects were exposed to 1 × 108 or 1 × 105 conidia/ mL, respectively ( Dalla Santa et al. 2009 ). Gomm et al. (2010) reported the efficacy of different dosages of the fungal formulation based on B. bassiana in the control of H. betulinus adults in field conditions. The treatments were 0, 2, 26, 50, 74, and 98 mL of the formulation based on B. bassiana CG 716 (Bovemax) with 1 × 107 conidia/mL. The doses of 50 and 98 mL were similar to each other and also were the only ones that were different from the control, though 50 mL has shown 33.26% of efficacy and the average time that the fungus took to kill the insect was 34.05 d. Leite et al. (2011) tested in laboratory several isolates of B. bassiana, B. brongniartii, M. anisopliae and Paecilomyces (= Isaria) sp. against H. betulinus adults. The concentration of fungal conidia applied was 3.5 × 107 conidia/mL in all cases. Mortality produced by B. bassiana varied between 66 to 100%, and their TL50 values varied from 9.8 to 26.4 d. These results were similar to ours, with a mortality rate between 51.1 and 86.3% and LT50 values between 9.6 and 13.6 d. However, Leite et al. (2011) observed that M. anisopliae and B. brongniartii caused 2.1 to 31.2% mortality and LT50 values from 17 to 25.8 d, differing from our results (mortality of 69.6 to 81.8% and LT50 from 7.4 to 7.9 d). In the previous report, *Paecilomyces* sp. produced 37.5 % mortality, whereas, in our study, the isolates of *P. lilacinum* were not pathogenic.

Our results indicate that different genera or species of entomopathogenic fungi have different pathogenicity. The median survival time can be attributed to various factors related to isolate infectivity and insect susceptibility. Virulence, infectivity, and pathogenicity are considered important properties of entomopathogens ( Casadevall and Pirofski 1999 , 2001; Thomas and Elkinton 2004 ). According to several authors preliminary records, entomopathogenic fungi infect insects to produce a large amount of secondary metabolites, including toxins attributed to pathogenicity, among which are low molecular weight compounds and other peptidic nature, as well as enzymes involved in the attack on the host ( Pucheta et al. 2006 , Borges et al. 2010 . Molnar et al. 2010 . Franco Chávez 2011 , Rohlfs and Churchill 2011 ).

Our results demonstrate a pathogenic effect of *B. bassiana* and *M. anisopliae sensu lato* on *H. betulinus* adults under laboratory conditions. Further research is necessary to determine the effectiveness of *B. bassiana* and *M. anisopliae* sensu lato under field conditions and to examine its potential impact on nontarget species.

## References

[R1] Abbott W. S . 1925 . A method of computing the effectiveness of an insecticide . *J. Econ. Entomol* . 18 : 265 – 267 .

[R2] Abdo C Nemer N. Nemer G Abou Jawdah Y. Kawar N. . 2008 . Isolation of *Beauveria* species from Lebanon and evaluation of its efficacy against the cedar web-spinning sawfly, *Cephalacia tannouriensis* . *Biocontrol* 53 : 341 – 352 .

[R3] Alencar F. R . 1960 . Erva-mate. *Serviço de Informação Agrícola* , pp. 10‒23. Rio de Janeiro .

[R4] Alves S. B . 1998 . *Controle microbiano de insetos* , 2 ed. Piracicaba, FEALQ São Paulo.

[R5] Angelo I. C. Fernandes E. K. K. Bahiense T. C. Perinotto W. M. S. Golo P. S. Moraes A. P. R. Bittencour V.R.E.P . 2012 . Virulence of *Isaria* sp. and *Purpureocillium lilacinum* to *Rhipicephalus microplus* tick under laboratory conditions . *Parasitol. Res* . 111 : 1473 – 1480 . 22710525 10.1007/s00436-012-2982-y

[R6] Bellows T. S . 2001 . Restoring population balance through natural enemy introductions . *Biol. Control* 21 : 199 – 205 .

[R7] Borges D Díaz A. O. San Juan A. N. Gómez E. . 2010 . Metabolitos secundarios producidos por hongos entomopatógenos. ICIDCA . *Sobre los Derivados de la Caña de Azúcar* 44 (3): 49-55.

[R8] Borges L. R . 2007 . *Eficiência de Beauveria bassiana (Bals.) Vuill. (Deuteromycota) para o controle de Hedypathes betulinus (Klug) (Coleoptera: Cerambycidae) em erva-mate, Ilex paraguariensis St.-Hil. (Aquifoliaceae),* p. 102 . Tese Doutorado em Entomol ogia-Universidade Federal do Paraná, Curitiba .

[R9] Borges L. R. Lázzari S. M. N. Lázzari F. A. . 2003 . Comparação dos sistemas de cultivo nativo e adensado de erva-mate, *Ilex paraguariensis* St.-Hil., quanto à ocorrência e flutuação populacional de insetos . *Rev. Brasil. Entomol.* 47 : 483-662.

[R10] Brownbridge M Reay S. D. Cummings N. J. . 2010 . Association of entomopathogenic fungi with exotic bark beetles in New Zealand pine plantations . *Mycopathologia* 169 : 75-80. 10.1007/s11046-009-9229-1 19669590

[R11] Butt T. M. Goettel. M. S. 2000 . Bioassays of entomogenous fungi, pp. 141-195. *In* A. Navon and K. R. S. Ascher (eds.). *Bioassays of entomopathogenic microbes and nematodes.* CAB International, Wallingford UK .

[R12] Casadevall A Pirofski L. . 1999 . Host-pathogens interactions: redefining the basic concepts of virulence and pathogenicity. *Infect. Immun.* 67 : 3703-3713. 10.1128/iai.67.8.3703-3713.1999 PMC96643 10417127

[R13] Casadevall A Pirofski L. . 2001 . Host-pathogen interactions: the attributes of virulence. *J. Infect. Dis.* 184 : 337-344. 10.1086/322044 11443560

[R14] Cassanello A. M. L . 1993 . *Ciclo de vida e aspectos morfológicos de Hedypathes betulinus (Klug, 1825) (Coleoptera: Cerambycidae), broca-da-erva-mate (Ilexparaguariensis St. Hil.,.* pp. 59. Dissertação (Mestrado em Entomologia - Curso de Pós-graduação em Ciências Biológicas) -Universidade Federal do Paraná, Curitiba

[R15] Dalla Santa H. S. Sousa N. J. Pittner E. Dalla Santa O. R. Soccol C. R. . 2009 . Controle biológico em pragas de *Ilex paraguariensis* (A. St.-Hil.) com fungo *Beauveria* sp . *Rev. Flor.* 39 : 67-76.

[R16] Dos Santos R. A Pozo N. M. . 2003 . Alternativa para el manej o de *Trialeurodes vaporariorum* Westwood en tomate orgánico en Uruguay . *Bol. Sanidad Vegetal. Plagas* 29 : 211-218.

[R17] Dube B Smart G. J . 1987 . Biological control of *Meloidogyne incognita* by *Paecilomyces lilacinus* and *Pasteuria penetrans* . J. Nematol. 19 : 222-227. PMC2618639 19290133

[R18] El Sayed G. N Ignoffo C. M. Leathers T. D. Gupta S. C. . 1993 . Cuticular and non-cuticular substrate influence on expression of cuticle-degrading enzymes from conidia of an entomopathogenic fungus, *Nomuraea rileyi* . Mycopathol. 122 : 79-87.

[R19] Ferron P . 1977 . Biological control of insect pests by entomogenous fungi . *Annu. Rev. Entomol.* 23 : 409-442.

[R20] Fiedler Z Sosnowska D. . 2007 . Nematophagous fungus *Paecilomyces lilacinus* (Thom) Samson is also a biological agent for control of greenhouse insects and mite pests. *BioControl* 52 : 547-558.

[R21] Franco Chávez K. G. Rodríguez Navarro S. Cervantes Mayagoitia J. F. Barranco Florido J. E. . 2011 . Enzimas y toxinas de hongos entomopatógenos, su aplicación potencial como insecticidas fungicidas . *Soc. Rural. Prod. Medio Ambiente* 11(22): 143-160.

[R22] Freimoser F. M. Screen S.S. Bagga S. Hu G. St Leger R.J. . 2003 . Expressed sequence tag (EST) analysis of two subspecies of *Metarhizium anisopliae* reveals a plethora of secreted proteins with potential activity in insect hosts . *Microbiolol.* 49 : 239-247. 10.1099/mic.0.25761-0 12576597

[R23] Glare T. R. Milner. R. J. 1991 . Ecology of entomopathogenic fungi, pp. 547-612. *In* D. K. Arora, K. G. Mukherji, and E. Drouhet (eds.) . *Handbook of applied mycology humans, animals, and insects.* Marcel Dekker, New York .

[R24] Glare T. R. Reay S. D. Nelson T. L. Moore R. . 2008 . *Beauveria caledonica* is a naturally occurring pathogen of forest beetles . *Mycol. Res.* 112 : 352-260. 10.1016/j.mycres.2007.10.015 18308525

[R25] Goettel M. S. Inglis G. D. Wraight S. P. . 2000 . Fungi, pp. 255-282. *In* L. A. Lacey and H. K. Kaya (eds.) . *Field manual in invertebrate pathology.* Kluwer Academic Press, Dordrecht, The Netherlands .

[R26] Gökçe A. Er M. K. . 2005 . Pathogenicity of *Paecilomyces* spp. to the glasshouse whitefly, *Trialeurodes vaporariorum,* with some observations on the fungal infection process . *Turk. J. Agric. For.* 29 : 331-339.

[R27] Gómez M. Tinti N. Alves L. . 1997 . Characterization of new biotypes of P157 strain of *Metarhizium anisopliae* var. *anisopliae,* got by treatment with gamma radiation . *Bol. Micol.* 12(2): 41-48.

[R28] Gomm P. C Furiatti R. S. Baranek E. Tlumaske L. Wagner F. O. . 2010 . Eficácia de diferentes dosagens do formulado fúngico à base de *Beauveria bassiana* (Vuill, 1912) no controle de adultos de *Hedypathes betulinus* (Klug, 1825) (Coleoptera: Cerambycidae) . *Rev. Acad. Ciên. Agrá. Ambien. Curitiba* 8(1): 55-60.

[R29] Hafez M Zaki F. N. Moursy A. Sabbour M. . 1994 . Biological effects of the entomopathogenic fungus, *Beauveria bassiana* on the Potato tuber moth *Pthorimaea operculella* (Seller) . *Anzeiger für Schädlingskunde, Pflanzenschutz, Umweltschutz* 70(8): 158-159.

[R30] Hajek A. E. St. Leger R. J. . 1994 . Interactions betwen fungal pathogens and insect hosts . *Annu. Rev. Entomol.* 39 : 293-322.

[R31] Headrick D. H. Groeden. R. D. 2001 . Biological control as a tool for ecosystem management . *Biol. Control* 21 : 249-257.

[R32] Hodge K. T . 2006 . *Clavicipitaceous anamorphs.* Dekker Reprint Program.

[R33] Humber R. A . 1991 . Fungal pathogens of aphids, pp. 45-56. *In* D. C. Peters, J. A. Webster, and C. S. Chlouber (eds.) . *Conference Proceedings.* Oklahoma St. Univ. Agric. Exp. Sta. MP 132 .

[R34] Humber R.A . 1996 . Fungi - Identification. *In* Lacey, L.A., editor. *Biological Techniques in Invertebrate Pathology,* pp.153-185 . Academic Press, London .

[R35] Humber R. A . 1997 . Fungi identification, pp. 153-185. *In* L. Lacey (ed.). *Manual of techniques in insect pathology.* Academic Press, London .

[R36] Inglis G. D. Goettel M. S. Butt T. M. Strasser H. . 2001 . Use of hyphomycetous fungi for managing insect pests, pp. 23‒69 . *In* T. M. Butt, C. Jackson, and N. Magan (eds.). *Fungi as biocontrol agents: progress problems and potential* . CABI Publishing, Oxfordshire, UK .

[R37] Lacey L. A. Frutos R. Kaya H. K. Vial P. . 2001 . Insect pathogens as biological control agents: do they have a future? *Biol. Control* 21 : 230 – 248 .

[R38] Lane B. S. Humphreys A. M. Thompson K Trinci A. P. J. . 1988 . ATP content of stored spores of *Paecilomyces farinosus* and the use of ATP as a criterion of spore viability . *Trans. Br. Mycol. Soc* . 90 : 109 – 148 .

[R39] Lanza L. M. Monteiro A. C. Malheiros E. B. . 2004 . População de *Metarhizium anisopliae* em diferentes tipos e graus de compactação do solo. *Ciên. Rural* 34 : 1757 – 1762 .

[R40] Leite M.S.P Soares E.T Iede Penteado S.C.R Castellano C. . 2000 . Seleção de linhagens de fungos entomopatogênicos para o controle de *Hedypathes betulinus* (Klug, 1825) (Coleoptera: Cerambycidae) em laboratório e eficiência da linhagem selecionada em campo . In: *Congresso sul-americano da erva-mate, 2., reunião técnica da erva-mate, Encantado, RS* . pp. 27-37 .

[R41] Leite M. S Iede P., E. T. Penteado, S. R. C. Zaleski S. R. M. Camargo J. M. M Ribeiro R. D. . 2011 . Seleção de isolados de fungos entomopatogênico para o controle de *Hedypathes betulinus* e avaliação da persistência . *Rev. Flor* . 41 : 619 – 628 .

[R42] Li Z. Z . 1988 . A list of insect hosts of *Beauveria bassiana* , pp. 241‒255. *In* Y.W. Li et al. (eds.) . *Study and application of entomogenous fungi in China* . Academic Periodical Press, Beijing .

[R43] Mendoza A. R. Sikora R. A. Kiewnick S. . 2007 . Influence of *Paecilomyces lilacinus* Strain 251 on the biological control of the burrowing nematode *Radopholus similis* in banana . *Neotropica* 37 : 203 – 213 .

[R44] Molnar I Gibson D. M. Krasnoff S. B. . 2010 . Secondary metabolites from entomopathogenic Hypocrealean fungi . *Nat. Prod. Rep* . 27 : 1233 – 1372 . 10.1039/c001459c 20601982

[R45] Monzón A . 2001 . Producción, uso y control de calidad de hongos entomopatógenos en Nicaragua. *Man. Integr. Plagas Agroecol* . 63 : 95 – 103 .

[R46] Núñez-Camargo M. C. Carrión G. Núñez-Sánchez A. E. López-Lima J. D. . 2012 . Evaluación de la patogenicidad in vitro de *Purpureocillium lilacinum* sobre *Globodera rostochiensis* . Trop. Subtrop. Agroecosys . 15 : S126‒S134.

[R47] Pagliosa M. M. dos Santos R. Diodato M. A. . 1994 . Patogenicidade do fungo entomopatogênico *Beauveria bassiana* (Bals.) Vuill. , em *Hedypathes betulinus* (Klug, 1825), praga da erva-mate, *Ilex paraguariensis* St.-Hil. *Agrárias Curitiba* . 13 : 229 – 231 .

[R48] Pendland J. C. Hung S. Y. Boucias D. G. . 1993 . Evasion of host defense by in vivo-produced protoplast-like cells of the insect mycopathogen *Beauveria bassiana* . *J. Bacteriol.* 175 : 5962 – 5969 . 8376342 10.1128/jb.175.18.5962-5969.1993 PMC206677

[R49] Posada F Marin M. Pérez S. . 1998 . *Paecilomyces lilacinus* enemigo natural de adultos de *Hypothenemus hampei* . *Cinecafé (Colômbia)* 49 : 72 – 77 .

[R50] Pucheta D. M. Flores A Rodríguez N. de la Torre M. . 2006 . Mecanismo de acción de los hongos entomopatógenos . *Interciencia.* 31 : 856 – 860 .

[R51] Ribeiro M. M. Santos H. R. Diodato M. A. . 1994 . Patogenicidade do fungo entomopatogênico *Beauveria bassiana* (Bals.). Vuill., em *Hedypathes betulinus* (Klug, 1825), praga da erva-mate, *Ilex paraguariensis* St.-Hill . *Rev. Setor Ciên. Agrár. Ambient.* 13 : 229 – 231 .

[R52] Rodriguez M. S. Gerding M. France A. . 2006 . Selección de aislamientos de hongos entomopatógenos para el control de huevos de la polilla del tomate *Tuta absoluta* Myrick (Lepidotera: Gelechiidae *)* . Agric. Téc . 66 : 151 – 158 .

[R53] Rohlfs M Churchill A. C. L. . 2011 . Fungal secondary metabolites as modulator of interactions with insects and other arthropods . *Fungal Genet. Biol* . 48 : 23 – 34 . 20807586 10.1016/j.fgb.2010.08.008

[R54] Samson R. A . 1974 . *Paecilomyces* and some allied hyphomycetes. *Stud. Mycol.* 6 : 1 – 119 .

[R55] Samson R. A. Evans H. C. Latge J. P. . 1988 . Atlas of entomopathogenic fungi . Springer-Verlag, Berlin .

[R56] Santoro P. H. Neves P. M. Alexandre T. M. Sartori D Alves L. F. Fungaro M. H. . 2008 . Selection of *Beauveria bassiana* isolates to control *Alphitobius diaperinus* . *J. Invertebr. Pathol* . 97 : 83 – 90 . 17869265 10.1016/j.jip.2007.07.009

[R57] Schenck S . 2004 . Control of nematodes in tomato with *Paecilomyces lilacinus* strain 251 . *Hawaii Agricultural Research Center Vegetable Report* 5 : 1 – 5 .

[R58] Shah P. A. Pell. J. K. 2003 . Entomopathogenic fungi as biological control agents . *Appl. Microbiol. Biotechnol* . 61 : 413 – 423 . 12764556 10.1007/s00253-003-1240-8

[R59] Shimazu M Zhang B. Liu Y. . 2002 . Fungal pathogens of *Anoplophora glabripennis* (Coleoptera: Cerambycidade) and their virulences . *Bull. FFPRI (For. For. Prod. Res. Inst.)* 1 : 1123 – 1130 .

[R60] Shin T. Y. Lee W. W. Ko1 S. H. Ji Z. Shin D. H. Son K. H. Park H. Y. Woo S. D. . 2011 . Preliminary evaluation of *Paecilomyces lilacinus* HY-4 to control *Tetranychus Urticae* . *Int. J. Ind. Entomol.* 22 : 25 – 28 .

[R61] St. Leger R. J. Frank D. C.. Roberts D. W Saples R. C. . 1992 . Molecular cloning and regulatory analysis of the cuticle degrading-protease structural gene from entomopathogenic fungus *Metarhizium anisopliae* . *Eur. J. Geochem.* 204 : 991 – 1001 . 10.1111/j.1432-1033.1992.tb16721.x 1551399

[R62] Suh E. G. Son K. H. Shin D. H. Kim K. D. . 2002 . Cultivation optimization of insect-pathogenic fungi *Paecilomyces lilacinus* HY-4 to soil-pest *Adoretus tenuimaculatus* . *Korean J. Entomol* . 32 : 133 – 139 .

[R63] Thomas S. R. Elkinton. J. S. 2004 . Pathogenicity and virulence . *J. Invertebr. Pathol* . 85 : 146 – 151 . 15109897 10.1016/j.jip.2004.01.006

[R64] Throne J. E. Weaker D. K. Chew V Baker J. E. . 1995 . Probit analysis of correlated data: Multiple observations over time at one concentration . *J. Econ. Entomol.* 88 : 1510 – 1512 .

[R65] Tscharntke T Klein A. M. Kruess A. Steffan-Dewenter I. Thies C. . 2005 .

[R66] Landscape perspectives on agricultural intensification and biodiversity—ecosystem service management . *Ecol. Lett* . 8 : 857 – 874 .

[R67] Tzean S. S. Hsieh L. S. Wu W. J. . 1997 . Atlas of entomopathogenic fungi from Taiwan . Council of Agriculture, Executive Yuan, Taipei, Taiwan .

[R68] Vu V. H. Hong S. I. Kim K. . 2007 . Selection of entomopathogenic fungi for aphid control . *J. Biosci. Bioeng* . 104 : 498 – 505 . 18215637 10.1263/jbb.104.498

[R69] Wakil W Ghazanfar M. U. Kwon Y. J. Ullah E. Islam S. Ali K. . 2012 . Testing *Paecilomyces lilacinus* , diatomaceous earth and *Azadirachta indica* alone and in combination against cotton aphid ( *Aphis gossypii* Glover) (Insecta: Homoptera: Aphididae) . *Afr. J. Biotechnol* . 11 : 821 – 828 .

[R70] Zimmerman G . 2007 . Review of the safety of the entomopathogenic fungi *Beauveria bassiana* and *Beauveria brongniartii* . Biocontrol Sci. Technol . 17 : 553 – 596 .

